# Motor imagery combined with brain-computer interface for stroke patients: a meta-analysis

**DOI:** 10.3389/fneur.2026.1672882

**Published:** 2026-01-20

**Authors:** Yuhuang Lin, Yong Yuan, Jingjing Chen, Xiangfu Lin

**Affiliations:** 1 Department of Rehabilitation, Sanya Hospital of Traditional Chinese Medicine (Hainan Hospital), Guangzhou University of Chinese Medicine, Sanya, Hainan, China; 2 Sanya City Yuan Yong Famous Doctor Studio, Sanya Hospital of Traditional Chinese Medicine (Hainan Hospital), Guangzhou University of Chinese Medicine, Sanya, Hainan, China; 3 Formulation Centre, Sanya Hospital of Traditional Chinese Medicine (Hainan Hospital), Guangzhou University of Chinese Medicine, Sanya, Hainan, China

**Keywords:** activities of daily living, brain-computer interface, meta-analysis, motor imagery, stroke, upper limb motor function

## Abstract

**Objective:**

To systematically evaluate the effects of motor imagery combined with brain-computer interface (MI-BCI) on stroke patients.

**Methods:**

Randomized controlled trials (RCTs) on MI-BCI for stroke patients were retrieved from CNKI, Wanfang, VIP, CBM, PubMed, Cochrane Library, Embase, and Web of Science databases from inception to June 2025. Data were analyzed using RevMan 5.2 software.

**Results:**

Eight RCTs involving 357 stroke patients were included. The meta-analysis showed that MI-BCI was associated with an improvement in upper limb motor function, although this did not reach conventional statistical significance (SMD = 0.86, 95% CI = −0.04 to 1.75, *p* = 0.06). In contrast, a statistically significant, moderate-to-large improvement was found in activities of daily living (SMD = 1.47, 95% CI = 0.51 to 2.44, *p* = 0.003). Subgroup analyses indicated that the efficacy in motor function was primarily evident when MI-BCI was administered as an adjunct to conventional rehabilitation or with an intervention duration of ≥4 weeks.

**Conclusion:**

The efficacy of MI-BCI is contingent upon its therapeutic context. When used as an adjunct to conventional rehabilitation, MI-BCI can significantly improve both upper limb motor function and activities of daily living in stroke patients. However, current evidence does not support its superiority over motor imagery alone when applied as a standalone therapy. An intervention duration of ≥4 weeks is recommended to achieve significant functional gains.

## Introduction

Stroke is one of the leading causes of long-term disability in adults worldwide, with approximately 50–70% of survivors experiencing varying degrees of upper limb motor dysfunction, significantly impairing their activities of daily living and quality of life (1, 2). Conventional rehabilitation methods, such as task-oriented training and constraint-induced movement therapy, can promote functional recovery but show limited efficacy in patients with severe motor impairments (3, 4). In recent years, research in neurorehabilitation has increasingly focused on novel intervention strategies based on neuroplasticity, among which Motor Imagery (MI) has garnered significant attention due to its ability to activate neural circuits similar to those involved in actual movement execution. MI enhances excitability in the motor cortex through mental simulation of limb movements, facilitating functional reorganization without relying on physical movement (5, 6). However, the clinical efficacy of MI alone remains contentious, as its benefits are highly dependent on the patient’s cognitive capacity and the quality of feedback, which is often subjective and unquantifiable. The emergence of Brain-Computer Interface (BCI) technology offers a promising solution to these limitations. A BCI system establishes a direct communication pathway between the brain and an external device. In the context of MI training, it acquires and decodes electroencephalogram (EEG) signals associated with motor intention in real-time. These decoded signals are then translated into intuitive visual, auditory, or tactile feedback (e.g., controlling a virtual limb), thereby creating a closed-loop system that provides objective, contingent feedback. This process is hypothesized to enhance neuroplasticity by reinforcing the activation of motor networks and boosting patient motivation and engagement (7). Consequently, several studies have reported that BCI-assisted MI training can significantly enhance upper limb motor function and activities of daily living in stroke patients (8).

Notwithstanding these promising reports, the literature presents inconsistent outcomes. Some randomized controlled trials have demonstrated notable improvements in Fugl-Meyer Assessment scores following MI-BCI intervention (8, 9), whereas others have found no statistically significant advantage over control interventions such as conventional therapy or MI alone (10, 11). This discrepancy may be attributed to variations in intervention parameters, patient characteristics (e.g., stroke chronicity), and BCI system performance. The existing heterogeneity underscores the necessity for a quantitative synthesis of current evidence to clarify the overall effect of MI-BCI on post-stroke recovery and to identify potential factors influencing its efficacy.

Furthermore, neuroimaging evidence suggests that MI combined with brain-computer interface (MI-BCI) training strengthens activation in the affected cerebral hemisphere and promotes functional reorganization in motor-related cortical areas (12). Given the heterogeneity and inconsistency in current research findings, coupled with the absence of a dedicated meta-analysis on this specific combined intervention, a systematic evaluation is urgently required. Therefore, this study aims to conduct a meta-analysis of randomized controlled trials to comprehensively assess the efficacy of MI-BCI on upper limb motor function and activities of daily living in stroke patients, and to explore the impact of intervention duration through subgroup analysis. The findings will provide higher-level evidence to inform clinical rehabilitation practice.

## Methods

### Inclusion and exclusion criteria

#### Inclusion criteria

(1) Study type: Randomized controlled trials (RCTs), with no restrictions on blinding. Publications were limited to those in Chinese or English.
(2) Participants: Patients diagnosed with stroke based on clinical and imaging examinations (13), with no restrictions on age, ethnicity, or geographic region.
(3) Interventions: The intervention group received MI-BCI intervention, which was defined as a closed-loop system where motor imagery is used to generate specific brain signals (e.g., sensorimotor rhythms) that are decoded by the BCI in real-time to trigger contingent feedback (e.g., movement of a virtual limb or functional electrical stimulation). The control group received conventional therapy, placebo, blank control, transcranial direct current stimulation (tDCS), MI alone, or BCI intervention alone.
(4) Outcome measures: Primary and Secondary outcomes.

Primary outcomes: Upper limb motor function, as measured by the Fugl-Meyer Assessment of the Upper Extremity (FMA-UE) score.
Secondary outcomes: Activities of daily living (ADL), as measured by the Modified Barthel Index (MBI). Included studies had to report data for at least one of the primary outcomes to be eligible for inclusion.

#### Exclusion criteria

(1) Studies with insufficient data, duplicate publications, or unavailable full texts.
(2) Reviews, conference abstracts, case reports, or animal studies.

### Search strategy

Systematic literature searches were performed from inception until June 2025 across the following electronic databases: China National Knowledge Infrastructure (CNKI), Wanfang Data, VIP Database, China Biology Medicine (CBM), PubMed, Cochrane Central Register of Controlled Trials (CENTRAL), Embase, and Web of Science Core Collection. To maximize search sensitivity, the strategy incorporated both Medical Subject Headings (MeSH) and relevant free-text terms for the key concepts: “Stroke,” “Motor Imagery,” and “Brain-Computer Interface.” The search strategies were customized for each database. The full electronic search strategies for all databases are provided in Supplementary File 1. Additionally, the reference lists of all included articles were manually screened to identify any potentially eligible studies not captured by the electronic search.

### Literature screening and data extraction

The literature screening process initially involved automatic deduplication using the NoteExpress computerized literature management system. After deduplication, preliminary screening was conducted by reviewing titles and abstracts. Subsequently, full-text articles were further examined to exclude those that did not meet the inclusion criteria. Following the search, all identified records were collated and uploaded into Reference Manager, and duplicates removed. The study selection process was carried out independently by two reviewers based on the pre-defined eligibility criteria. First, titles and abstracts were screened, and then the full texts of potentially relevant studies were retrieved and assessed in detail. Any disagreements were resolved through discussion or by consulting a third reviewer. The data extraction form was piloted on one included study. Data extraction from the included studies was then performed independently by the same two reviewers. For studies reporting data as median with interquartile range or other non-parametric formats, we estimated the sample mean and standard deviation using established methods (14) to allow for meta-analysis. Similarly, for data reported as mean with implausibly small variability (suspected standard error), we converted the values to estimated standard deviations based on sample size.

### Quality assessment of literature

Two researchers independently evaluated the quality of the included studies according to the criteria outlined in the Cochrane Handbook for Systematic Reviews of Interventions (Version 5.1.0) (15). The assessment covered the following aspects: Whether the study described the specific method and process of random sequence generation; Whether the study described the method of allocation concealment; Whether the study prevented participants and personnel from knowing the intervention assignments; Whether outcome assessors were blinded to the intervention; Whether the study reported complete outcome data, including any missing data; Whether the study selectively reported outcomes (either positive or negative); Other potential sources of bias. Studies that fully met the criteria were rated as Grade A; those that partially met the criteria were rated as Grade B; and those that did not meet the criteria at all were rated as Grade C. In case of disagreement between the two researchers, a third researcher was consulted to reach a consensus.

### Statistical methods

The statistical analysis was performed using RevMan 5.2 software. The I^2^ test was employed to assess clinical heterogeneity. When *p* ≥ 0.05 and I^2^ < 50%, it indicated no significant statistical heterogeneity among the studies, and a fixed-effects model was applied for the meta-analysis. Conversely, if statistical heterogeneity was present, the sources of heterogeneity were first analyzed. If no apparent clinical differences were identified and no definitive statistical source of heterogeneity could be determined, a random-effects model was used for the meta-analysis. The Fugl-Meyer Upper Extremity Motor Function and Modified Barthel Index included in this study were both continuous variables; thus, the standardized mean difference (SMD) and its 95% confidence interval (CI) were used as the effect measures. A *p*-value < 0.05 was considered statistically significant.

## Results

### Basic characteristics and quality assessment of included studies

**Figure 1:**
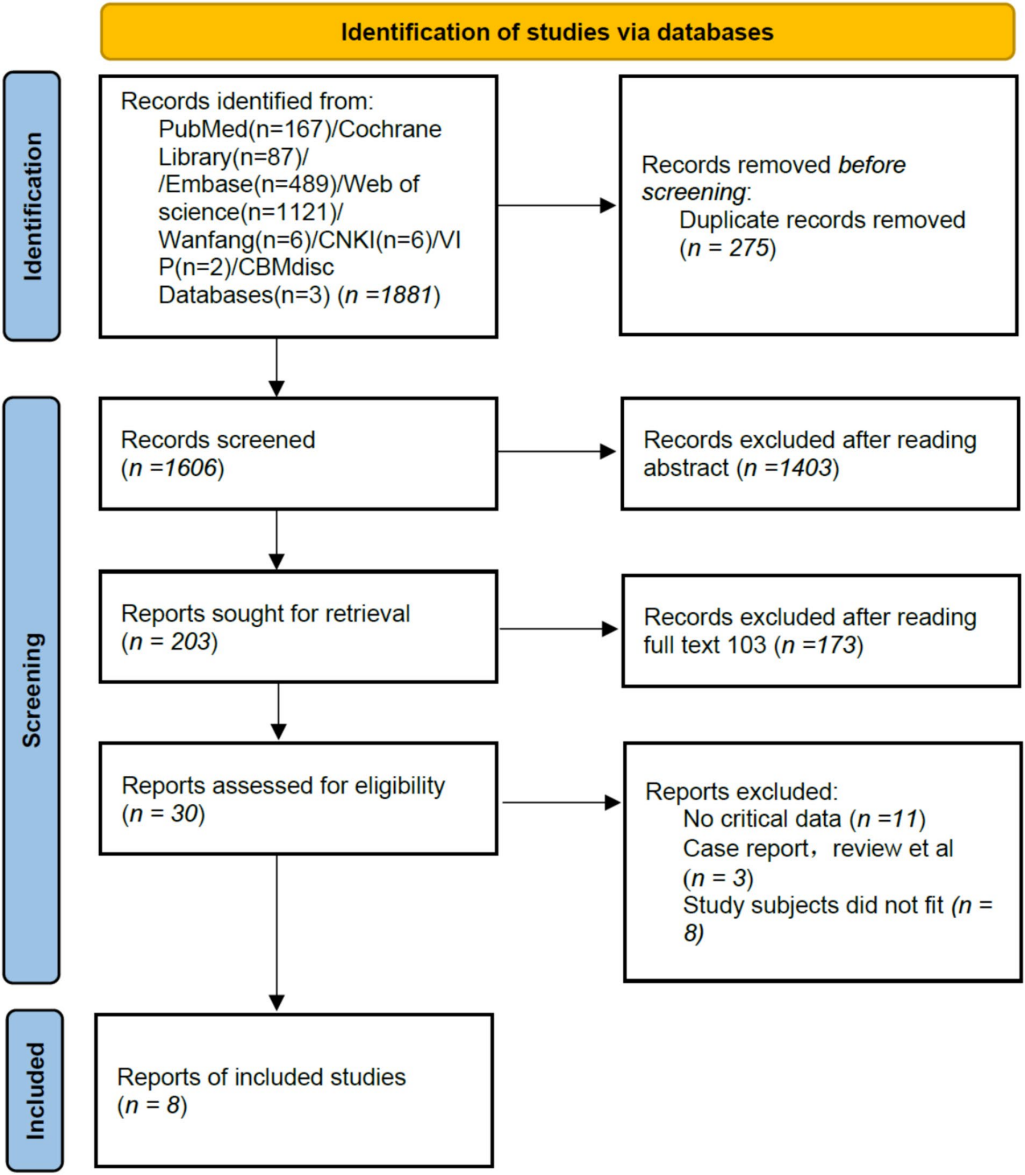
Literature screening flowchart.

A total of 1,881 articles was retrieved from the database search, and ultimately, 8 studies (9–11, 16–20) were included. The literature screening process is shown in Figure 1.

### Basic characteristics and methodological quality assessment of included studies

**Figure 2:**
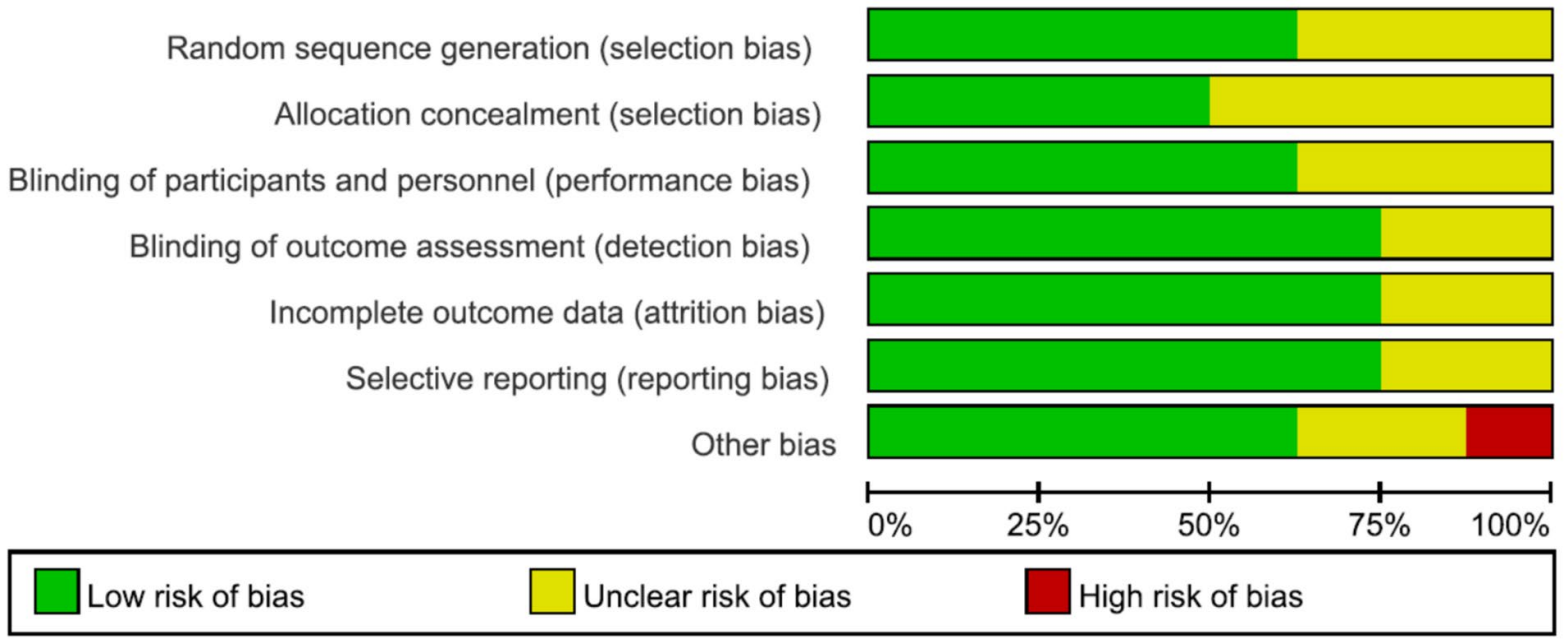
Risk of bias assessment graph for included literature.

A total of 8 English-language studies (9–11, 16–20) were included, involving 357 stroke patients (178 in the intervention group and 179 in the control group). The basic characteristics of the studies are presented in Table 1. Quality assessment of the included studies revealed 5 Grade A studies and 3 Grade B studies. The risk of bias assessment graph for the included studies is shown in Figure 2.

**Table 1:** Basic characteristics of included literature.

Author and year	Country	Stroke type	Sample size		Age		Intervention method		Treatment duration	Single intervention duration	Outcome measures	Sessions per week
			Control group	Intervention group	Control group	Intervention group	Intervention group	Control group				
Liao 2023 (10)	Turkey	Ischemic stroke	20	20	61.00 ± 3.70	61.50 ± 3.80	MI-BCI + Conventional treatment	Conventional treatment	3 weeks	45–60 min	①	5
Li 2022 (16)	China	Ischemic and hemorrhagic stroke	12	12	55.00 ± 12.20	43.80 ± 14.70	MI-BCI + Conventional treatment	Conventional treatment	2 weeks	3 h	①②	5
Ming 2025 (17)	China	Subacute stroke (ischemic and hemorrhagic)	14	15	60.79 ± 11.48	56.13 ± 8.63	MI-BCI	Transcranial direct current stimulation (tDCS)	4 weeks	30 min	①②	5
Hu 2021 (11)	China	Ischemic and hemorrhagic stroke	43	44	60.40 ± 16.80	44.90 ± 7.50	MI-BCI	MI	19 days	30 min	①	Not Reported
Kim 2025 (9)	South Korea	Ischemic and hemorrhagic stroke	13	12	46.00 ± 12.80	49.00 ± 16.90	MI-BCI	MI	4 weeks	60 min	①	5
Luo 2024 (18)	China	Acute ischemic stroke	32	32	Not Reported	Not Reported	MI-BCI + Conventional treatment	Conventional treatment	2 weeks	60 min	②	10
Pichiorri 2015 (19)	Italy	Unilateral cortical, subcortical, or mixed stroke	14	14	Not Reported	Not Reported	MI-BCI	MI	4 weeks	30 min	①	3
Liu 2023 (20)	China	Ischemic and hemorrhagic stroke	30	30	53.0 (38.5, 59.5) years [median (Q1, Q3)]	52.5 (45.0, 59.3) years [median (Q1, Q3)]	MI-BCI + Conventional treatment	Conventional treatment	3 weeks	20 min	①②	5

MI-BCI, Motor Imagery Brain-Computer Interface; MI, Motor Imagery; ① Fugl-Meyer Assessment of the Upper Extremity (FMA-UE) score; ② Modified Barthel Index (MBI).

### Meta-analysis results

**Figure 3:**
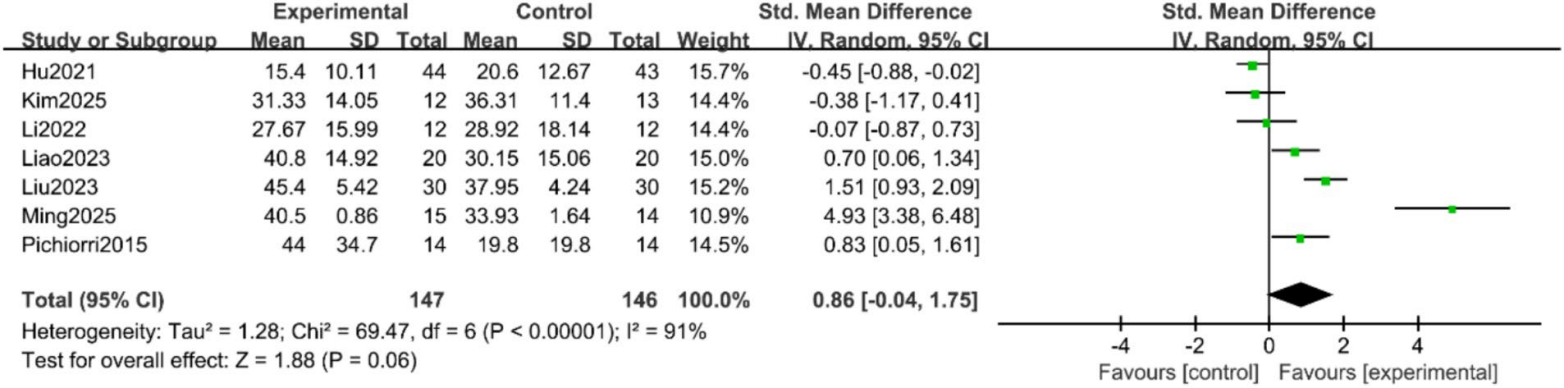
Forest plot of the meta-analysis for FMA-UE scores.

**Figure 4:**
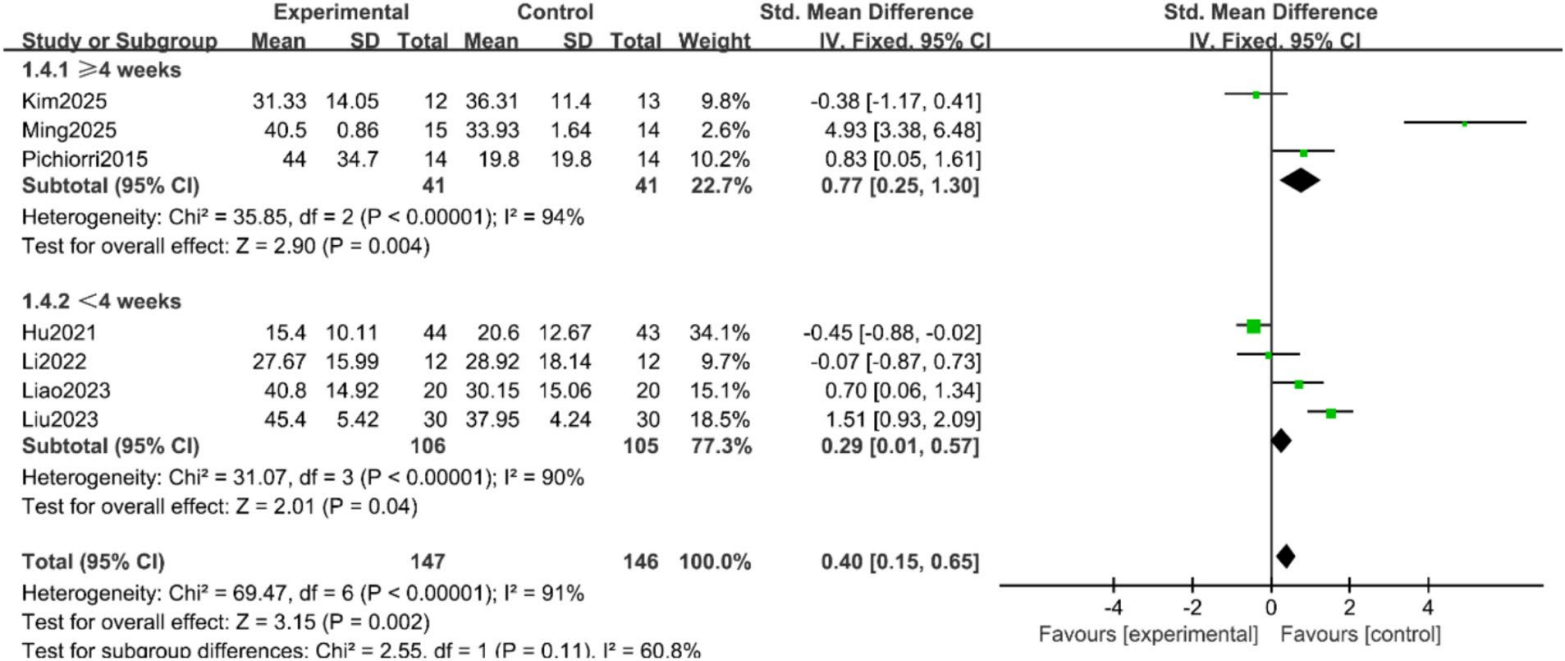
Forest plot of the subgroup analysis by treatment duration for FMA-UE scores.

**Figure 5:**
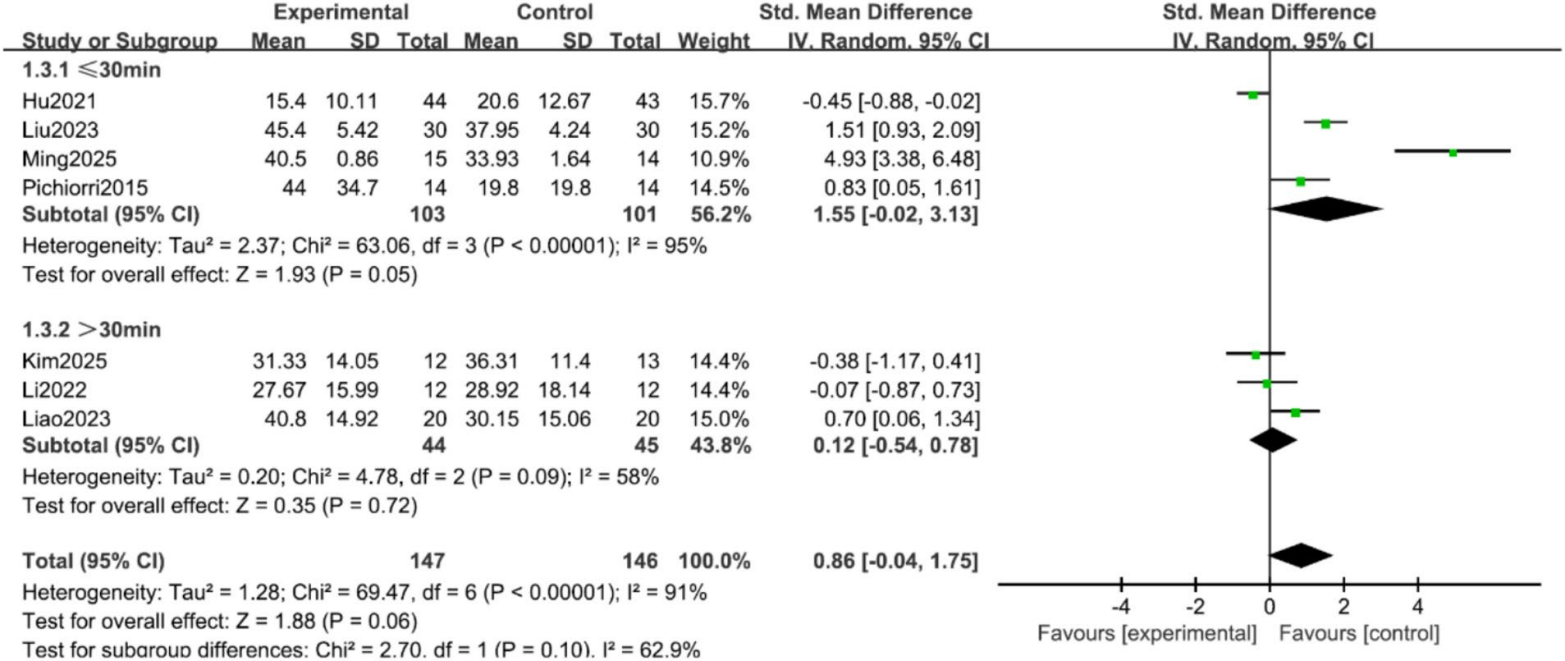
Forest plot of the subgroup analysis by single-session duration for FMA-UE scores.

FMA-UE Scores: Seven studies (9–11, 16, 17, 19, 20) reported the FMA-UE scores of patients, with significant heterogeneity among the studies (*p* < 0.00001, I^2^ = 91%). A random-effects model was applied after sensitivity analysis. The results indicated no statistically significant difference in FMA-UE scores between the two groups (SMD = 0.86, 95% CI = −0.04 to 1.75, *p* = 0.06), as shown in Figure 3. Subgroup analyses were conducted based on treatment duration and single-session intervention time. To address the potential heterogeneity arising from varied control and experimental interventions, pre-specified subgroup analyses were performed according to: (1) the nature of the control group (conventional rehabilitation, motor imagery alone, or other active interventions); and (2) the composition of the experimental intervention (MI-BCI as a standalone therapy vs. MI-BCI combined with conventional rehabilitation). The results suggested that when the treatment duration was ≥4 weeks, the intervention group showed superior FMA-UE scores compared to the control group (SMD = 0.77, 95% CI = 0.25 to 1.30, *p* = 0.004). For treatment durations <4 weeks, the difference remained statistically significant, albeit with a smaller effect size (SMD = 0.29, 95% CI = 0.01 to 0.57, *p* = 0.04) (Figure 4). Additionally, no statistically significant differences was found for the subgroups with single-session intervention times ≤30 min (SMD = 1.55, 95% CI = −0.02 to 3.13, *p* = 0.05), nor for the >30 min subgroup (SMD = 0.12, 95% CI = −0.54 to 0.78, *p* = 0.72) (Figure 5).

**Figure 6:**
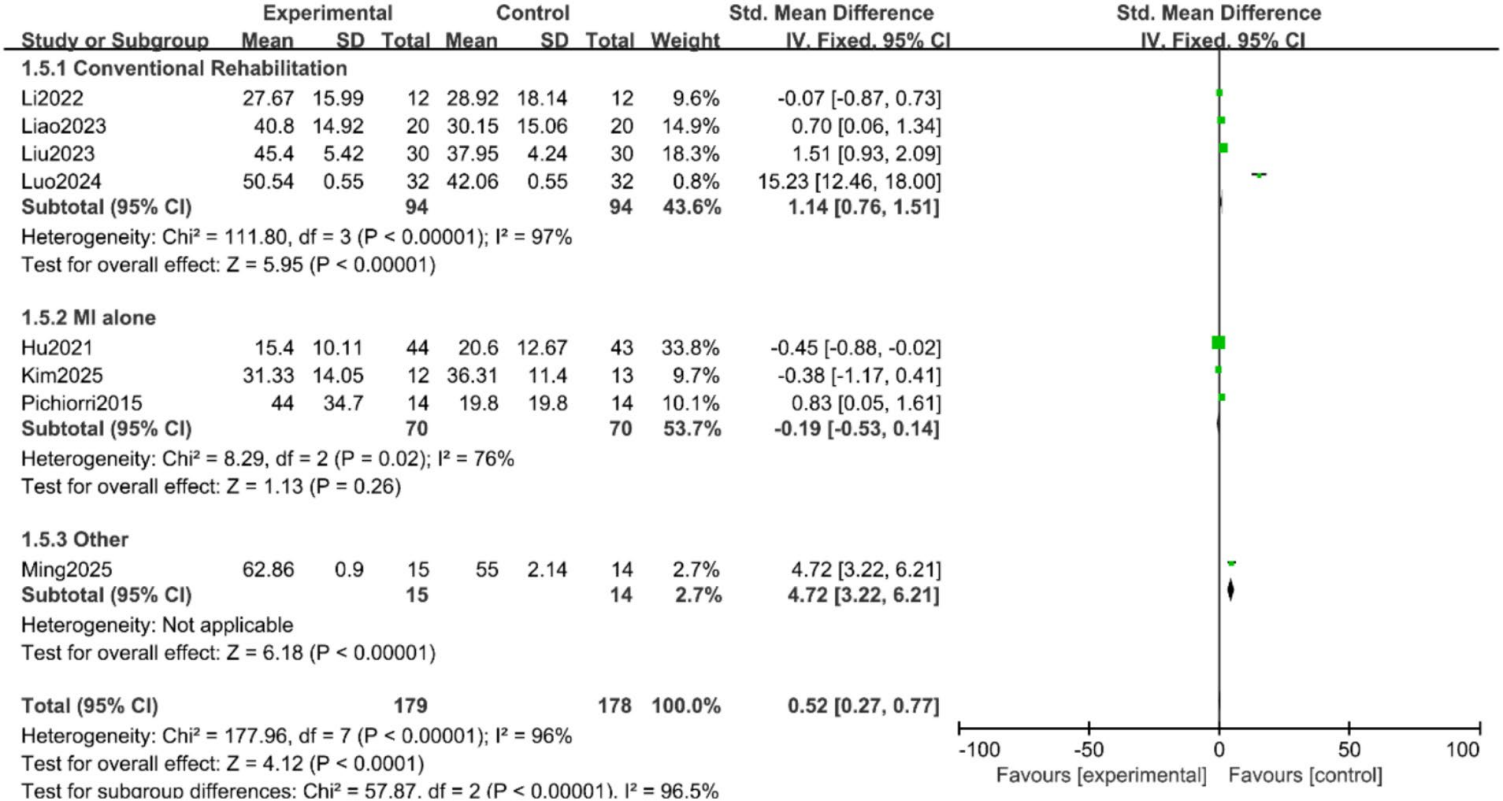
Forest plot of the subgroup analysis for control group type.

Analysis by control group type: The effect of MI-BCI was highly dependent on the control intervention. A large and statistically significant effect was observed when MI-BCI was compared to conventional rehabilitation alone (SMD = 1.14, 95% CI 0.76 to 1.51, *p* < 0.00001). In stark contrast, no significant benefit was found when MI-BCI was compared to motor imagery (MI) alone (SMD = −0.19, 95% CI −0.53 to 0.14, *p* = 0.26). The test for subgroup differences was statistically significant (*p* < 0.00001, I^2^ = 96.5%), confirming that the control group type is a major source of heterogeneity (Figure 6).

**Figure 7:**
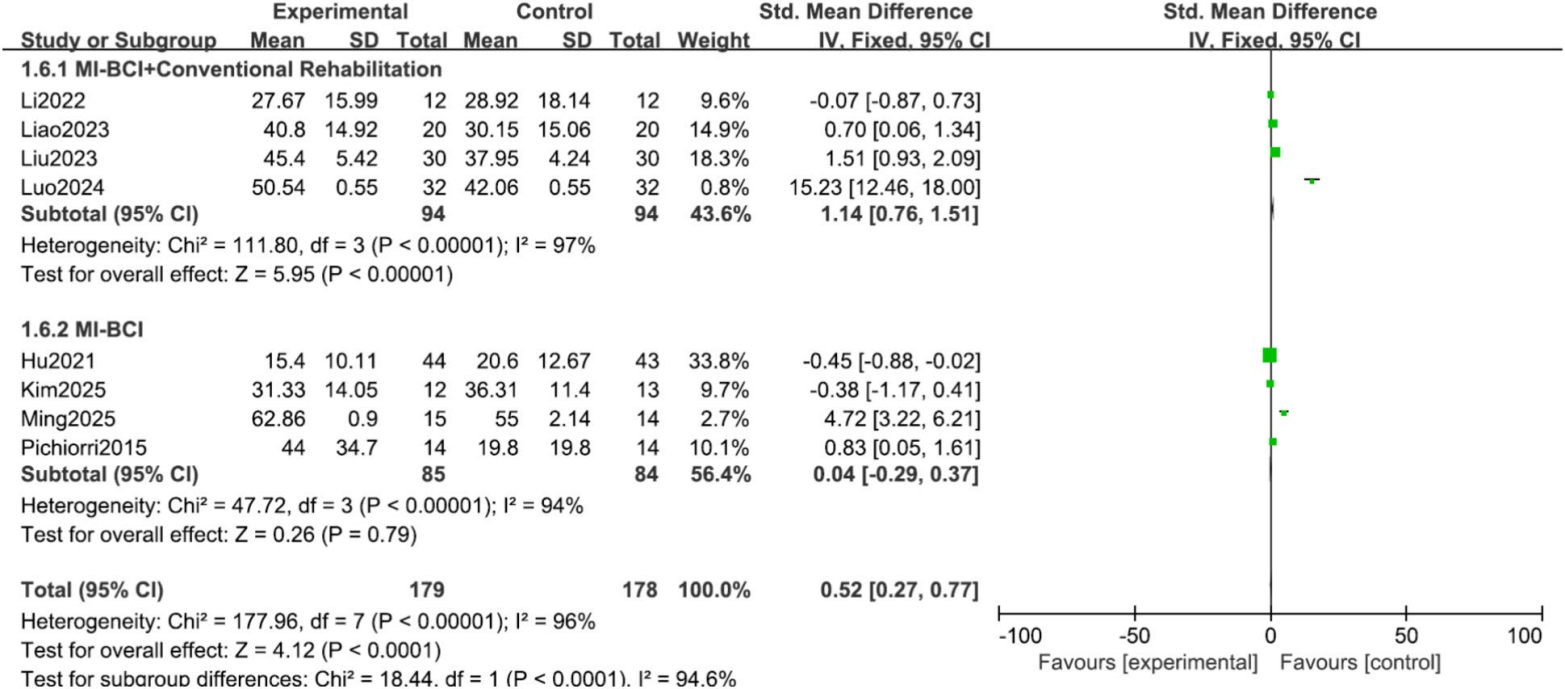
Forest plot of the subgroup analysis for experimental group purity.

Analysis by experimental group purity: The overall effect was exclusively driven by the combined intervention approach. The MI-BCI combined with conventional rehabilitation subgroup showed a large and significant effect (SMD = 1.14, 95% CI 0.76 to 1.51, *p* < 0.00001). Conversely, MI-BCI alone as a standalone therapy showed no significant effect (SMD = 0.04, 95% CI −0.29 to 0.37, *p* = 0.79). The test for subgroup differences was significant (*p* < 0.00001, I^2^ = 94.6%), indicating that the presence of concomitant conventional therapy is a critical effect modifier (Figure 7).

**Figure 8:**
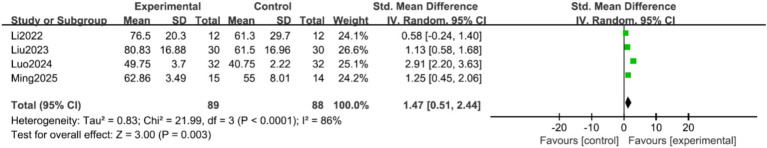
Forest plot of the meta-analysis for modified Barthel index (MBI).

Activities of daily living: activities of daily living: Four studies reported MBI scores (16–18, 20). To address data reporting issues, we applied conversions to ensure compatibility for meta-analysis. For studies (16, 18), which reported implausibly small variances (suggestive of standard error rather than standard deviation) and median (interquartile range) data respectively, we estimated the mean and standard deviation using established methods (see Methods). The meta-analysis of the four studies with converted data showed that the MI-BCI intervention led to a moderate-to-large and statistically significant improvement in activities of daily living compared to the control group (SMD = 1.47, 95% CI = 0.51 to 2.44, *p* = 0.003) (Figure 8).

## Discussion

This meta-analysis revealed that the overall improvement in Fugl-Meyer Upper Extremity Scores following MI-BCI training in stroke patients did not reach statistical significance, which is inconsistent with the findings of Rodríguez-García et al. (21). This discrepancy may be attributed to the following factors: First, the time-dependent nature of neuroplasticity may play a critical role. Recent studies suggest that functional reorganization of the motor cortex post-stroke requires cumulative training duration to induce significant changes (22). The average intervention period in the included trials was relatively short (2–4 weeks), which may have been insufficient to elicit lasting neural remodeling. Second, patient heterogeneity may have obscured the intervention effects. The enrolled studies included stroke patients ranging from acute to chronic stages, with significant variability in neuroplastic potential. For instance, subacute-phase patients may exhibit greater sensitivity to BCI training, whereas chronic-phase patients, whose neural reorganization has largely stabilized, may require longer-term interventions. Additionally, the technical limitations of BCI systems cannot be overlooked. Some studies employed EEG signal decoding with limited accuracy, potentially leading to delayed or imprecise feedback, thereby diminishing the efficacy of closed-loop training (23).

The subgroup analysis revealed that patients in the intervention group with a treatment duration of ≥4 weeks showed significantly better Fugl-Meyer Upper Extremity Scores compared to those with <4 weeks. This outcome may stem from the following neurophysiological mechanisms: BCI reinforces neural plasticity in the affected sensorimotor cortex by decoding motor-related EEG signals in real time. Recent studies indicate that MI-BCI training significantly increases blood-oxygen-level-dependent (BOLD) signals in the affected primary motor cortex and supplementary motor area (24), which aligns with the superior outcomes observed in the longer-duration subgroup in this study. This suggests that neural remodeling requires cumulative stimulation. Furthermore, BCI translates motor intentions into visual or tactile feedback (e.g., virtual hand movement), forming an “intention-feedback” closed loop that mimics the perception-action coupling process of actual movement. Such closed-loop training may activate the mirror neuron system, facilitating motor learning and corroborating the reinforcing effects of neurofeedback (25). Notably, subgroup analyses based on single-session duration did not show significant effects. This may be because 30 min is already sufficient to induce acute neuromodulatory effects, or because exceeding 30 min could lead to fatigue, offsetting additional benefits (26). This finding carries important clinical implications: priority should be given to ensuring the sustained duration of MI-BCI intervention (≥4 weeks) rather than merely extending single-session training time. Clinical treatment protocols should adopt a “high-frequency, moderate-to-long duration” approach (e.g., 3–5 sessions per week for 4–6 weeks) to accumulate sufficient neuroplasticity stimulation. Single-session duration may be maintained at 30–45 min—sufficient to induce effective neuromodulation while avoiding patient fatigue.

This meta-analysis demonstrates that MI-BCI significantly improves activities of daily living (ADL) in stroke patients, which is consistent with the findings of Liu et al. (20). The MI-BCI training tasks integrate BCI feedback with daily activities, directly training functional movements. This task-oriented design may facilitate the integration of motor planning and actual function by activating the dorsal visual stream and the parietal-premotor cortical network (27). Furthermore, the real-time feedback provided by BCI enhances patients’ self-efficacy and training compliance. Relevant studies have found that the completion rate of training sessions in the MI-BCI group was significantly higher than that in the MI alone group (19), which may indirectly improve participation in daily living activities. MI-BCI serves as an effective supplementary approach to traditional ADL training. Future research could further expand its application scope by developing intelligent home-based MI-BCI training systems.

This meta-analysis revealed significant clinical heterogeneity, which was decisively explained by our additional subgroup analyses. The most striking finding is that the efficacy of MI-BCI is contingent upon its clinical context. The large, significant effect observed when MI-BCI is added to conventional rehabilitation (SMD = 1.14), compared to its lack of effect as a standalone therapy against MI alone (SMD = −0.19), provides a crucial clarification for the field.

This pattern suggests that the primary value of MI-BCI in stroke rehabilitation may not be as a monolithic, stand-alone intervention, but rather as a powerful adjunct or facilitator within a broader, conventional rehabilitation framework. The BCI component likely enhances patient engagement, provides goal-oriented practice, and reinforces motor learning in a way that synergizes with conventional techniques. The non-significant effect of MI-BCI alone indicates that the BCI-driven feedback, by itself in the studied doses and forms, may not be sufficient to surpass the effects of well-structured mental practice alone. This underscores that future research and clinical implementation should focus on optimizing how MI-BCI is integrated into existing rehabilitation protocols, rather than viewing it as a competing alternative.

This study has several limitations. First, the sample size was limited, with only seven studies involving 357 patients included. The enrolled populations comprised both acute and chronic stroke phases, and control interventions were heterogeneous (e.g., conventional rehabilitation, motor imagery alone, tDCS). Second, there were methodological shortcomings: only four studies detailed random sequence generation, none achieved blinding of participants and therapists, and three did not clearly report allocation concealment. Third, significant clinical heterogeneity was present, stemming from variations in intervention protocols, patient characteristics, and outcome measures. Additionally, to enable meta-analysis, we converted data from studies reporting median (with interquartile range) or suspected standard error into mean and standard deviation, which may introduce estimation error. Fourth, although stroke chronicity is a key potential effect modifier, a prespecified subgroup analysis was not feasible due to inconsistent reporting in the original studies. Fifth, as most trials were short-term, the long-term effects of MI-BCI remain unclear. Finally, due to the small number of included studies, we did not perform sensitivity analyses (e.g., leave-one-out), as such analyses would be underpowered and produce unstable results. Future meta-analyses with more primary studies should incorporate these methods to test robustness. Further high-quality trials with standardized reporting, longer follow-up, and detailed patient stratification are needed to confirm and extend these findings.

This meta-analysis demonstrates that the impact of Motor Imagery combined with Brain-Computer Interface (MI-BCI) on post-stroke recovery is not uniform but is profoundly influenced by its mode of application. The primary conclusion is that MI-BCI serves as a highly effective adjunctive therapy rather than a standalone intervention. The robust improvement in upper limb motor function and activities of daily living is predominantly observed when MI-BCI is integrated with conventional rehabilitation, suggesting a synergistic effect where BCI-enhanced mental practice amplifies the benefits of physical training.

Conversely, the evidence does not currently establish that MI-BCI alone provides a significant advantage over carefully administered motor imagery training without BCI feedback. This indicates that the real-time feedback loop, while crucial, may function best as a catalyst within a comprehensive rehabilitation program. Furthermore, the achievement of significant functional outcomes is dependent on a sufficient intervention dose, with a duration of ≥4 weeks proving most effective.

Therefore, for clinical practice, we recommend the implementation of medium- to long-term (≥4 weeks) MI-BCI protocols as a complement to, not a replacement for, conventional stroke rehabilitation. Future research should prioritize optimizing these combined protocols and identifying patient characteristics that predict the best response to MI-BCI augmented therapy.

## Data Availability

The original contributions presented in the study are included in the article/Supplementary material, further inquiries can be directed to the corresponding author/s.
