# Supplementary material for: Motor imagery combined with brain-computer interface for stroke patients: a meta-analysis
Source: Front Neurol. 2026 Jan 20;17:1672882. doi: 10.3389/fneur.2026.1672882 (PMC12866895; doi:10.3389/fneur.2026.1672882)
Supplement: Supplementary file 1 [file Table_1.DOCX]

**Supplementary File 1: Search Strategies**

**Databases and Search Strings:**

1. **CNKI:**
   SU=('运动意象') AND SU=('脑机接口') AND SU=('脑卒中')
2. **VIP:**
   M=(运动意象) AND M=(脑机接口) AND M=(脑卒中)
3. **Wanfang:**
   主题:(运动意象) and 主题:(脑机接口) and 主题:(脑卒中)
4. **CBM:**
   ("运动意象"[常用字段:智能]) AND ("脑机接口"[常用字段:智能]) AND ("脑卒中"[常用字段:智能])
5. **PubMed:**

("Stroke"[Mesh] OR "Cerebrovascular Accident"[Title/Abstract] OR "Post-Stroke"[Title/Abstract] OR "Hemiplegia"[Title/Abstract]) AND

("Motor Imagery"[Title/Abstract] OR "Movement Imagery"[Title/Abstract] OR "Kinesthetic Imagery"[Title/Abstract] OR "Mental Practice"[Title/Abstract] OR "Imagery, Psychomotor"[Mesh]) AND

("Brain-Computer Interfaces"[Mesh] OR "BCI"[Title/Abstract] OR "Brain Machine Interface"[Title/Abstract] OR "Brain-Machine Interface"[Title/Abstract])

1. **Cochrane Library:**
   (Motor Imagery):ti,ab,kw AND (Brain-Computer Interface):ti,ab,kw AND (stroke):ti,ab,kw
2. **Web of Science:**
   TS=("Motor Imagery") AND TS=("Brain-Computer Interface" OR "Brain Machine Interface") AND TS=(stroke)
3. **Embase (via Ovid):**

'motor imagery'/exp OR 'motor imagery' OR (motor AND imagery)

AND

'brain-computer interface'/exp OR 'brain-computer interface' OR (brain-computer AND interface)

AND

'stroke'/exp OR stroke
